# Impact of Overweight in Mens with Family History of Hypertension: Early Heart Rate Variability and Oxidative Stress Disarrangements

**DOI:** 10.1155/2020/3049831

**Published:** 2020-07-30

**Authors:** Ariane Viana, Danielle da Silva Dias, Mario Cesar Nascimento, Fernando dos Santos, Fernanda de Cordoba Lanza, Maria Cláudia Irigoyen, Kátia De Angelis

**Affiliations:** ^1^Translational Physiology Laboratory, Universidade Nove de Julho (UNINOVE), Sao Paulo, Brazil; ^2^Department of Physiology, Federal University of Sao Paulo, Sao Paulo, Brazil; ^3^State University of Santa Catarina (UDESC), Santa Catarina, Brazil; ^4^Heart Institute (InCor), University of São Paulo, Medical School, Sao Paulo, Brazil; ^5^Department of Physiotherapy, Federal University of Minas Gerais-UFMG, Belo Horizonte, MG, Brazil

## Abstract

**Aim:**

To evaluate cardiovascular, autonomic, and oxidative stress markers in eutrophic and overweight offspring of hypertensive parents comparing them to eutrophic and overweight offspring of normotensive parents.

**Methods:**

We conducted a cross-sectional study. We selected 71 male and sedentary subjects, divided into 4 groups: eutrophic group with a negative family history of hypertension (EH-, *n* = 18) or positive family history of hypertension (EH+, *n* = 17), overweight group with a negative family history of hypertension (OH-, *n* = 19) or a positive family history of hypertension (OH+, *n* = 17), and aged between 18 and 35 years.

**Results:**

Blood glucose was higher in the OH+ group when compared to other groups. Diastolic blood pressure was increased in OH- and OH+ groups when compared to eutrophic groups. Regarding the HRV, the LF abs was higher in OH- and OH+ groups when compared to the EH- group. LF/HF values were higher in EH+ and OH+ groups when compared to the EH- and OH- groups. As to oxidative stress and the metabolism of nitric oxide, we observed an increase in hydrogen peroxide and nitrite levels in the OH+ group, and in the NADPH oxidase in OH- and OH+ groups when compared to the other groups.

**Conclusion:**

Our findings demonstrate that the overweight group with a family history of hypertension presented all the dysfunctions observed in isolation from these risk factors. We observed an exacerbation of cardiac sympathetic modulation and early prooxidants increase, which may be associated with target organ damage and cardiovascular risk in this population.

## 1. Introduction

Cardiovascular diseases (CVD) are the leading cause of mortality from noncommunicable diseases, accounting for 29.6% of deaths worldwide [1]. In Brazil, epidemiological data show alarming rates, with 30% deaths attributable to CVDs [2]. The contribution of genetic factors to the genesis of hypertension is well established in the literature [3, 4]. However, there are no genetic variants, which may be used to predict individual risk factors for hypertension [3]. The onset of hypertension depends on the interaction between genetic predisposition and environmental factors [1]. In this sense, we have been hypothesized that genetic variations may contribute to the determination of an individual's blood pressure (BP) levels. Several family studies have demonstrated the familial aggregation of arterial hypertension, both among siblings, and between parents and children [5]. Some researchers have argued that having a family history of hypertension doubles the individual's risk of developing hypertension, regardless of other factors, such as weight, age, or smoking [6].

A number of studies assessing the variability of the cardiac R-R intervals have found that individuals with a positive family history of hypertension at rest have increased sympathetic and decreased cardiac parasympathetic activity when compared to individuals with no family history of hypertension [7, 8] (SAHA et al., 2015). In addition, sympathetic modulation has been found to be increased in prehypertensive subjects [8], and a significant increase in diastolic BP has been demonstrated in healthy normotensive individuals with a family history of arterial hypertension [9]. Furthermore, genetic predisposition to hypertension has been associated with a greater central fat deposition [10].

Obesity has been pointed out as a risk factor for several cardiovascular and metabolic diseases, such as diabetes mellitus type 2, hypertension, dyslipidemia, and acute myocardial infarction [11]. In addition, CVD mortality has been found to increase considerably with elevated BP [2]. The relationship between cardiovascular, metabolic, and sympathetic dysfunction has been demonstrated in both animal and human studies [12, 13].

Given these risk factors, and the well-established association between lifestyle and the increased prevalence of overweight and obesity, classical and new mechanisms underlying the onset of this dysfunction need a fuller understanding. In this sense, the primary purpose of the present study was to evaluate cardiovascular, autonomic, and oxidative stress markers in eutrophic and overweight offspring of hypertensive parents by comparing them to eutrophic and overweight offspring of normotensive parents. In the secondary aim of this study, we investigated these subjects regarding anthropometric, glycemic, and lipid profiles. We tested the hypothesis that overweight children of hypertensive parents would present more striking early dysfunctions than those of the eutrophic offspring of hypertensive parents.

## 2. Materials and Methods

This is a cross-sectional analytical study, approved by the Ethics Committee of the Universidade Nove de Julho (UNINOVE) under registration number 1.672.599. We selected 71 male subjects (UNINOVE students) from March/2016 to March/2017. They were divided them into 4 groups: an eutrophic group with a negative family history of hypertension (EH-, *n* = 18), an eutrophic group with a positive family history of hypertension (EH+, *n* = 17), an overweight group with a negative family history of hypertension (OH-, *n* = 19), and an overweight group with a positive family history of hypertension (OH+, *n* = 17).

The study included male individuals aged between 18 and 35 years, with a body mass index (BMI) between 18.5 and 29.99 Kg/m^2^ classified according to the Brazilian Guidelines for Obesity [14]. The subjects classified according to the International Physical Activity Questionnaire (IPAQ) as physically active, individuals with BMI as obese type I or above, smokers with deceased or unknown parents, or parents under 50, were excluded from the study. We also excluded subjects using medications, which may interfere with BP levels, such as thyroid hormones, nonsteroidal anti-inflammatory drugs, antidepressants, and illicit drugs, as well as those with conditions which may trigger secondary hypertension, such as diseases of the blood vessels, kidneys, and endocrine glands.

**Figure 1:**
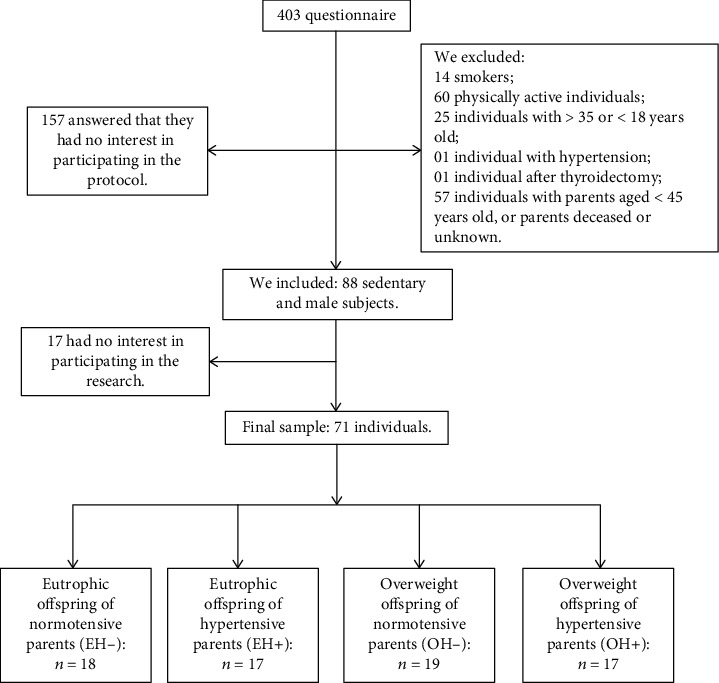
Study design.

The individuals of this study were selected among students from Universities. After students were fully informed on the study, 403 questionnaires were sent to classrooms for the first contact and selection of individuals. We initially recruited 88 individuals for this research. After the first contact, 17 individuals did not show interest in participating in the research for a range of reasons, e.g., lack of time or schedule incompatibility, and our final sample totaled 71 individuals (Figure 1). Individuals were selected through a questionnaire evaluating family history of hypertension and other diseases (which has been proven by viewing your parents' average prescription and/or medications in use), body mass, and height, as well as the subjects' availability for participation in the study. All subjects signed an Informed Consent Form (TCLE) before answering the questionnaire.

The level of physical activity was evaluated by the IPAQ [15]. Only subjects classified as sedentary (0) or insufficiently active (1) were included in the study. The assessment of the body composition was performed by a qualified professional and was undertaken by calculating body mass index (BMI), abdominal circumference (AC), performed at the umbilical scar level [16], waist circumference (WC), performed from the midpoint of the iliac crest to the last rib [16, 17], and hip circumference (HC), evaluated in the greater trochanter [17]. Skinfold measurements were carried out using an adipometer (Cescorf®, at a constant pressure of 10 g/mm^2^) and were always performed on the right side of the body. After collecting the skinfolds of the triceps and subscapular region, we added the three measurements and averaged them. Three nonconsecutive measurements of the folds were performed, as established in the Slaughter protocol [18]. We performed the calculation stipulated in the protocol to obtain the body fat value of individuals. In addition, body composition by the method of electrical bioimpedance with Biodynamics Model 450 Tetrapolar Body Composition Analyzer. To perform this procedure, the individuals were instructed to fast for 12 hours, abstaining from caffeine, alcohol, and exercise for at least 24 hours, not ingesting water for four hours and emptying their bladder 30 minutes before the procedure. Evaluation was performed with the individual in dorsal decubitus. One emitter electrode was placed close to the metacarpal phalangeal joint of the dorsal surface of the right hand and another one distally from the transverse arch of the upper surface of the right foot. Moreover, one detector electrode was placed between the distal prominences of the radius and ulna of the right wrist and another one between the medial and lateral malleoli of the right ankle, following the manufacturer's recommendations.

Blood pressure was measured in the sitting position by the auscultatory method, using an aneroid cuff sphygmomanometer, following the guidelines of the Brazilian Society of Hypertension and the ESC/ESH guidelines for the management of arterial hypertension [19, 20], and a stethoscope for BP measurement. The assessment of cardiac autonomic modulation was performed by recording the RR interval, using the Polar® heart rate monitor (RS800 model). The RR interval was recorded over the 20-minute rest period of each individual and was evaluated in the time and frequency domains. The spectrum resulting from the Fast Fourier Transform (FFT) modeling is derived from all recorded signal. It includes the entire signal variance, regardless of whether its frequency components appear as specific spectral peaks or as nonpeak broadband power. The RMSSD (root mean square of the successive differences of the RR interval) reflects the variability in the change in the NN interval. Spectral power for low (LF: 0.03–0.15 Hz) and high (HF: 0.15–0.4 Hz) frequency bands, representative of cardiac sympathetic and parasympathetic modulation, respectively, was calculated by means of power spectrum density integration within each frequency bandwidth, using a customized routine (Cardioseries) [21].

After a 12-hour fast, a blood sample was collected from the subjects of the research at the Nove de Julho University for the assessment of hematological and biochemical parameters for the determination of glucose, total cholesterol, triglycerides, HDL, LDL, VLDL, and glycated hemoglobin. These parameters were then analyzed by the AFIP Laboratory (Associação Fundo de Incentivo à Pesquisa) associated with the Nove de Julho University.

Additionally, plasma samples were collected for the determination of oxidative stress. NADPH oxidase enzyme activity was measured in plasma. For the assay, a 50 mM phosphate buffer containing 2 mM EDTA and 150 mM sucrose, NADPH, 3 mM, and 10 *μ*l of sample were used [22]. Hydrogen peroxide was measured by radish peroxidase (HRP) mediated by phenol red oxidation, leading to the formation of a measurable compound at 630 nm. We performed a curve with Distilled H_2_O_2_ 250 *μ*M, Radish Peroxidase Solution (PRS), composed of Buffer Dextrose, Phenol Red (Sigma-Aldrich Corporation), and Type II Radish Peroxidase (Sigma-Aldrich Corporation), and Sodium Hydroxide (NaOH) (FMaia Gold). Plasma was added to the 70 *μ*l ELISA Pate, along with 180 *μ*l PRS and then incubated for 25 min at room temperature. After this period, 5 *μ*l of NaOH was added and read on an ELISA plate reader [23]. Plasma nitrite levels were measured by the reaction of the samples with the microplate Griess reagent (96 wells) in an ELISA reader. Total nitrite was obtained by the reaction of the samples with a 50 *μ*l Griess reagent. Total nitrite was estimated from a standard absorbance curve at 592 nm [24].

### 2.1. Statistical Analysis

The sample size calculation was performed using the G ∗ Power 3 software (Düsseldorf, Bundesland, Germain). Considering that the RR variance is representative of the total HRV, it is widely used in clinical studies and is associated with cardiovascular outcomes [21]; we used the RR variance as the main outcome for sample size calculation. Through a pilot study with the actual sample of this study, with four groups, with a standard deviation of the RR variance between the 1300 groups, with an effect size of 0.40, assuming an alpha error of 0.05 and an error of 0.10 beta (90%), the total sample size was 68 individuals divided into four groups, i.e., 17 per group. Due to possible sample losses, 20% of individuals in each group were added, totaling 20 in each group, and a total of 80 individuals in the sample.

Statistical analysis was performed by the Graph Prism® software. The results are presented as mean ± standard deviation of the mean. The data were tested for normality and were found to be parametric by the Shapiro-Wilk test. Statistical analysis of the data was performed using the two-way ANOVA followed by Bonferroni post hoc. The significance level adopted was *p* < 0.05.

In order to interpret the magnitude of change in parameters at different time points, the “effect size” (ES) was measured. Based on statistical literature, the interpretation of this index was compiled, namely, small ES = 0.10, medium ES = 0.25, and large ES = 0.40. When expressing the findings of a quantitative study, ES is important because a value can identify if there is an effect but cannot reveal its magnitude [25].

## 3. Results

The groups showed no difference in age, as displayed in Table 1. Individuals in the overweight groups (OH- and OH+) presented higher values of body mass, and AC when compared to the eutrophic groups (EH- and EH+). The OH+ group presented lower lean mass values when compared to the eutrophic groups (Table 1). Cardiovascular risk, assessed by the waist-to-hip ratio, presented higher values in the overweight groups (OH- and OH+) when compared to the eutrophic groups (EH- and EH+, *p* ≤ 0.0041) (Table 1). As expected, OH- (26.76 ± 1.77 kg/m^2^) and OH+ (29.96 ± 3.52 kg/m^2^) had higher BMI values when compared to the EH+ group (22.4 ± 1.99 kg/m^2^) and EH- group (22.39 ± 1.69 kg/m^2^) (*p* < 0.0001; ES = 1.00). Similar differences were observed regarding skinfolds, which showed a higher percentage of fat mass in the OH- (26.11 ± 7.44%) and OH+ (32.04 ± 12.57%) groups when compared to EH- group (18.23 ± 6.03%) (*p* < 0.0001; ES = 0.99). As to fat mass, a difference was found between the OH+ and EH+ groups (18.85 ± 5.10%) (*p* < 0.0001; *ES* = 0.99). When we evaluated the body composition of these individuals, using bioimpedance, we observed lower fat mass values in the EH- (20.08 ± 5.47%) and EH+ (20.01 ± 3.58%) groups when compared to the OH+ group (27.58 ± 4.97%, *p* < 0.0001; ES = 0.98).

Through the IPAQ, we found that our *sample* was exclusively *composed* of sedentary (= 0) or insufficiently active individuals (= 1). We did not observe any difference between groups in our assessment of the physical activity level (*p* = 0.26). Similarly, there was no difference among the groups in mean physical activity time (minutes/week) (*p* = 0.38). Regarding blood biochemical profile, there was no difference between the groups in the parameters evaluated. However, the blood glucose, presented higher values in the OH+ group than in the EH- group (*p* = 0.04; *ES* = 0.41), as shown in Table 2.

**Figure 2:**
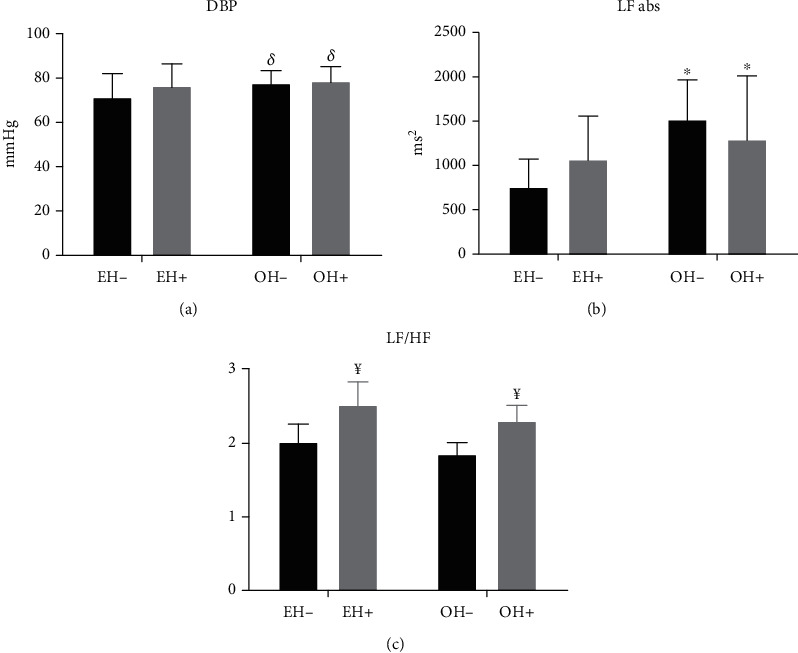
(a) Diastolic blood pressure (DBP); (b) low frequency (LF) bands in absolute values; (c) Simpathovagal balance (LF/HF) of RR interval in studied groups. ^*δ*^*p* = 0.03 vs. absence of overweight. ^∗^*p* = 0.0001 vs. EH-. ^¥^*p* = 0.04 vs. absence history of hypertension; EH-: eutrophic offspring of normotensive parents; EH+: eutrophic offspring of hypertensive parents; EH-: overweight offspring of normotensive parents; EH+: overweight offspring of hypertensive parents.

Regarding cardiovascular parameters, we did not observe any differences in SBP (*p* = 0.61) and HR (*p* = 0.11) in the studied groups (Table 3). However, the groups with the overweight factor, OH- (77.6 ± 5.62 mmHg) and OH+ (78.4 ± 6.79 mmHg), showed an increase in DBP when compared to the eutrophic groups, EH- (71.2 ± 10.67 mmHg), and EH+ (76.4 ± 9.88 mmHg) (*p* = 0.0287; ES = 0.84), as shown in Figure 2(a).

After recording the RR interval, we performed a linear analysis of HRV. We found no differences between the groups in total variance, standard deviation (SD), RMSSD (Root Mean Square of the Successive Differences), and in HF and LF normalized values, as shown in Table 3. When we evaluated the absolute LF of pulse interval (PI), we observed a significant increase in the OH+ group (1303 ± 721 ms^2^) and OH- (1527 ± 450 ms^2^) when compared to the EH- group (764 ± 317 ms^2^) (*p* < 0.05; ES = 0.99), as shown in Figure 2(b). There was no difference for the absolute HF values in the studied groups (EH- group: 529 ± 226 ms^2^; EH+ group: 662 ± 518 ms^2^; OH- group: 499 ± 193 ms^2^; OH+ group: 553 ± 289 ms^2^; *p* = 0.37; *ES* = 0.33). For LF/HF values, groups with a positive family history of hypertension presented higher values when compared to groups with a negative family history (EH+: 2.52 ± 1.0 and OH+: 2.30 ± 0.80 vs. EH-: 2.01 ± 0.84 and OH-: 1.85 ± 0.67, *p* = 0.04, ES = 0.82), as shown in Figure 2(c).

**Figure 3:**
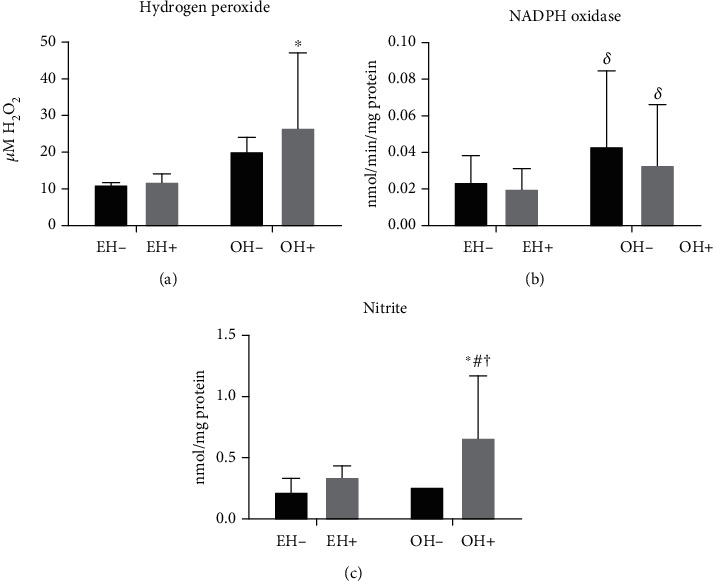
(a) Hydrogen peroxide concentration; (b) NADPH oxidase activity; (c) nitrate levels in plasma of the studied groups. ^∗^*p* = 0.003 vs. EH-. ^*δ*^*p* = 0.04 vs. EH- and EH+. E# *p* = 0.02 vs. EH+; ^†^*p* = 0.001 vs. FNS. EH-: eutrophic offspring of normotensive parents; EH+: eutrophic offspring of hypertensive parents; OH-: overweight offspring of normotensive parents; OH+: overweight offspring of hypertensive parents.

Concerning the oxidative stress, we observed an increase in hydrogen peroxide concentration (*p* = 0.003; ES = 0.99) in the OH+ group (26.5 ± 20.4 *μ*M H_2_O_2_) when compared to the other groups - EH- (11.1 ± 2.08 *μ*M H_2_O_2_), EH+ (11.9 ± 0.61 *μ*M H_2_O_2_), and OH- (20.2 ± 16.9 *μ*M H_2_O_2_) (Figure 3(a)). As to NADPH oxidase, there was an increase in values (*p* = 0.03; ES = 0.89) in the overweight groups, OH- (0.043 ± 0.04 (nmol/min/mg protein) and OH+ (0.033 ± 0.03 (nmol/min/mg protein) when compared to the eutrophic factor in the EH- (0.023 ± 0.01 (nmol/min/mg protein) and EH+ groups (0.020 ± 0.011 (nmol/min/mg protein) (Figure 3(b)). Regarding nitric oxide *bioavailability*, there was an increase in plasma nitrite levels (*p* = 0.01, ES = 0.99) in the OH+ group (0.66 ± 0.51 nmol/mg protein) when compared to OH- (0.26 ± 0.13 nmol/mg protein), EH- (0.22 ± 0.11 nmol/mg protein) and EH+ groups (0.34 ± 0.09 nmol/mg protein), as shown in Figure 3(c).

## 4. Discussion

High BP increases the risk of CVD for millions of people worldwide [1] and a family history of hypertension doubles the risk for the onset of this condition. In addition, studies have shown early cardiometabolic changes in offspring of hypertensives [6], which may be exacerbated by modifiable risk factors, such as sedentary lifestyle and obesity. In this sense, the present study aimed at evaluating the mechanisms underlying the early dysfunctions observed in the offspring of hypertensive individuals with and without overweight.

As a main result, we observed a significant increase in cardiac sympathetic modulation accompanied by an increase in DBP in the overweight groups (OH- and OH+) when compared to the eutrophic groups (EH- and EH+). Also, we found a significant increase in cardiac sympathovagal balance in the groups with a family history of hypertension (EH+ and OH+) when compared to individuals with a negative family history of hypertension (EH- and OH-). Regarding metabolic alterations, we observed an increase in blood glucose only when we compared the OH+ to the EH- group. Overweight (OH- and OH+ groups) was associated with increased NADPH oxidase activity. However, hydrogen peroxide and nitric oxide nitrite values were only increased in the group with all risk factors (OH+).

In our study, we did not observe either age differences or significantly different levels of physical activity (minutes/week) in the groups studied, thus characterizing our sample as young and sedentary adults. Additionally, we observed that, as expected, the OH- and OH+ groups presented higher BMI when compared to the EH- and EH+ groups. However, a few studies have shown that only the BMI is a slightly refined parameter for determining body fat values. In this sense, we evaluated other variables, and we found a significant increase in AC, an indication of abdominal fat deposition and a marker of higher cardiometabolic risk [26] in the OH+ and OH- groups when compared to the EH- and EH+ groups. In fact, AC has been shown to be an index as effective as the WC/HC ratio to assess cardiovascular risk [27]. WC/HC also demonstrated a significant increase in overweight individuals in our study, even though these values remain within the normal range.

A key finding of our study lies in the increase in DBP in the overweight groups (OH- and OH+) when compared to the eutrophic groups. In this sense, some studies have shown a relationship between higher values of fat mass and a worsening of autonomic modulation and higher BP [27, 28]. However, other studies have shown increased risk for CVDs in individuals with a positive family history, and with overlapping factors, such as obesity, overweight, and sedentary lifestyle [29].

Regarding HRV, our linear analysis demonstrated that overweight individuals (OH- and OH+) presented increased sympathetic modulation, represented by the absolute LF band of PI when compared to the EH- group. Regarding LF/HF, an increase in this parameter was observed in the groups with a family history of hypertension (EH+ and OH+) when compared to EH- and OH- groups. These findings corroborate previous studies indicating worse autonomic modulation in individuals with a family history of hypertension. These studies have found an increase in sympathetic modulation associated with a reduction in PI variance at rest [7, 9, 29–31] and in young individuals with prehypertension [32]. In addition, data from the literature have pointed that sympathovagal balance is worsened in the offspring of hypertensive parents after carbohydrate intake, which may be associated with increased risk for cardiovascular events, including early death [33]. A recent study involving young, active, and sedentary women has found that a sedentary lifestyle impairs autonomic response after mental stress [34].

It should be mentioned that findings from the literature have shown that hypertension alone may increase oxidative stress and vice versa [32]. A study has demonstrated a strong correlation between oxidative stress and vagal modulation and prehypertension, suggesting that oxidative stress may play a crucial role in the pathogenesis of hypertension [32]. In our study, we observed a systemic increase of NADPH oxidase activity and the concentration of hydrogen peroxide in overweight individuals (OH- and OH+). In this sense, obesity seems to be also associated with oxidative stress. The accumulation of adipose tissue may stimulate the production of ROS in the adipose cells through the activation of NADPH oxidase [35]. It seems clear that excess fat in adipocytes increases the activity of NADPH oxidase, reduces the activity of antioxidant enzymes (superoxide dismutase and catalase), and attracts leukocytes to the adipose tissue. Both pathways generate EROs, such as hydrogen peroxide (H_2_O_2_), and promote oxidative stress through protein and lipid oxidation [36].

Our findings suggest that in the study population, offspring of hypertensive and/or overweight individuals have early alterations of oxidative stress mediators, such increased NADPH oxidase (EH- and EH+ *vs.* OH- and OH+) and hydrogen peroxide (OH+ *vs.* EH-, EH+, and OH-), although we have not yet observed increased markers of oxidative damage (lipoperoxidation or protein oxidation). This may be due to the fact that we evaluated young adults who were still normotensive.

Along with this, the increased levels of nitrite in individuals with a family history of hypertension and overweight found in our study corroborate with a previous study. MARSEGLIA et al. (2014) have also observed an increase in oxidative stress in such individuals and a higher risk for hypertension due to the formation of peroxynitrite and the reduction of vasodilation. Thus, it is possible that the HRV dysfunction may signal an alteration of inflammatory mediators and oxidative stress, the so-called anti-inflammatory cholinergic reflex [37]. This induces cardiometabolic worsening (still within normal range) in sedentary young adults with a family history of hypertension.

Our findings demonstrate that sedentary young adults with a family history of hypertension and/or overweight present impairment in HRV. However, the major finding of our study lies in the fact that the overweight group with a family history of hypertension presented all the dysfunctions observed in isolation from these risk factors. This occurred regardless of increased BP or exacerbation of alterations in the metabolic profile. The subjects also presented an exacerbation of nitric oxide metabolism and cardiac sympathetic modulation, which may be associated with increased target organ damage and cardiovascular risk in this population. Therefore, lifestyle modifications, such as physical activity and weight loss, may play a critical role in lowering the risk for cardiovascular events in this population, which, though young and apparently healthy, show early changes associated with potential cardiovascular risk. It should be emphasized that studies relating genetic factors to modifiable environmental factors in a young and healthy population may provide data on noninvasive therapeutic tools and offer informed strategies to prevent the onset of CVDs, thus reducing mortality from hypertension, obesity, metabolic syndrome, and associated conditions.

## Figures and Tables

**Table 1:** Anthropometric assessments in studied groups.

	EH- (*n* = 18)	EH+ (*n* = 17)	OH- (*n* = 19)	OH+ (*n* = 17)	*p*	ES
Age (years)	25.5 ± 6.35	25.8 ± 5.11	26.5 ± 5.33	25.5 ± 4.25	0.390	0.07
Weight (kg)	69.7 ± 7.12	69.3 ± 9.86	81.1 ± 7.57^∗^^#^	93.0 ± 11.81^∗^^#†^	0.002	1.00
Height (m)	1.76 ± 0.04	1.75 ± 0.07	1.74 ± 0.05	1.76 ± 0.04	0.682	0.19
AC (cm)	74.2 ± 7.10	75.2 ± 9.31	88.5 ± 7.14^∗^#	95.1 ± 11.48^∗^#	<0.0001	1.00
WC/HC	0.79 ± 0.04	0.79 ± 0.04	0.85 ± 0.05^∗^^#^	0.87 ± 0.06^∗^^#^	0.002	0.74
Lean mass (%)	78.2 ± 7.25	79.3 ± 4.23	76.6 ± 4.69	72.4 ± 4.97^∗^#	0.014	0.48

^∗^ *p* < 0.05 vs. EH-; ^#^*p* < 0.05 vs. EH+; ^†^*p* < 0.05 vs. OH-. EH-: eutrophic offspring of normotensive parents; EH+: eutrophic offspring of hypertensive parents; OH-: overweight offspring of normotensive parents; OH+: overweight offspring of hypertensive parents. ES: effect sizes AC: abdominal circumference; WC/HC: ration waist circumference and hip circumference.

**Table 2:** Biochemical parameters in studied groups.

	EH- (*n* = 18)	EH+ (*n* = 17)	OH- (*n* = 19)	OH+ (*n* = 17)	*p*	ES
Blood glucose (mg/dL)	82.3 ± 5.14	83.5 ± 9.09	86.6 ± 4.76	89.0 ± 5.88^∗^	0.016	0.41
Urea (mg/dL)	31.1 ± 6.23	29.1 ± 5.30	28.9 ± 5.66	31.2 ± 7.81	>0.999	0.18
Creatinine (mg/dL)	0.92 ± 0.14	0.89 ± 0.08	0.96 ± 0.11	0.89 ± 0.12	0.434	0.25
Total cholesterol (mg/dL)	151 ± 36.5	162 ± 27	166 ± 37.6	179 ± 30.9	0.095	0.30
HDL (mg/dL)	48.9 ± 9.2	51.0 ± 11.1	48.2 ± 10.6	43.1 ± 14.8	0.853	0.24
LDL (mg/dL)	83.3 ± 22.1	92.6 ± 25.2	98.2 ± 30.6	111.5 ± 30.3^∗^^#†^	<0.0001	0.37
Triglycerides (mg/dL)	96.2 ± 75.7	92.4 ± 30.4	81.5 ± 28.6	109.0 ± 39.4	0.528	0.22
VLDL (mg/dL)	20.0 ± 14.7	18.5 ± 6.03	16.2 ± 5.6	21.8 ± 7.9	0.458	0.09
Glycated hemoglobin (%)	5.3 ± 0.32	5.2 ± 0.37	5.1 ± 0.30	5.4 ± 0.35	0.517	0.33

^∗^ *p* < 0.05 vs. EH-; ^#^*p* < 0.05 vs. EH+; ^†^*p* < 0.05 vs. OH EH-: eutrophic offspring of normotensive parents; EH+: eutrophic offspring of hypertensive parents; OH-: overweight offspring of normotensive parents; OH+: overweight offspring of hypertensive parents. ES: effect sizes.

**Table 3:** Cardiovascular and heart rate variability parameters in the studied groups.

	EH- (*n* = 18)	EH+ (*n* = 17)	OH- (*n* = 19)	OH+ (*n* = 17)	*p*	ES
SBP (mmHg)	115.5 ± 10.5	118.0 ± 10.1	119.9 ± 5.7	120.2 ± 8.6	0.344	0.21
HR (bpm)	77.0 ± 10.7	72.3 ± 8.1	71.8 ± 7.8	74.4 ± 9.6	0.633	0.22
Variance PI (ms^2^)	2947 ± 635	2916 ± 1402	3163 ± 767	3677 ± 2261	0.957	0.23
SD (ms)	53.9 ± 6.0	52.5 ± 13.1	56.1 ± 6.0	58.3 ± 17.1	0.635	0.20
RMSSD (ms)	36.2 ± 8.8	33.2 ± 10.1	36.1 ± 7.2	35.1 ± 14.5	0.241	0.21
LF nu (%)	60.9 ± 8.3	65.2 ± 9.3	58.3 ± 8.9	63.6 ± 9.05	>0.999	0.29
HF nu (%)	38.9 ± 8.2	34.5 ± 9.2	41.4 ± 9.07	36.0 ± 9.1	0.441	0.30

SBP: Systolic Blood Pressure; HR: heart rate; SD: standard deviation; RMSSD: Root Mean Square of the Successive Differences; LF nu: low-frequency band, normalized data; HF nu: high-frequency band, normalized data; EH-: eutrophic offspring of normotensive parents; EH+: eutrophic offspring of hypertensive parents; OH-: overweight offspring of normotensive parents; OH+: overweight offspring of hypertensive parents. ES: effect sizes.

## Data Availability

The oxidative stress assessment and RR interval data used to support the findings of this study are available from the corresponding author upon request.
